# *catena*-Poly[[bis­(1-methyl-1*H*-imidazole-κ*N* ^3^)zinc]-μ-3-nitro­phthalato-κ^2^ *O* ^1^:*O^2^*]

**DOI:** 10.1107/S1600536812004576

**Published:** 2012-02-10

**Authors:** Xi-Juan Zhang, Ming-Lin Guo

**Affiliations:** a School of Materials and Key Laboratory of Hollow Fiber Membrane Materials and Membrane Process, Tianjin Polytechnic University, Tianjin 300387, People’s Republic of China; b School of Environment and Chemical Engineering, and Key Laboratory of Hollow Fiber Membrane Materials and Membrane Process, Tianjin Polytechnic University, Tianjin 300387, People’s Republic of China

## Abstract

In the title complex, [Zn(C_8_H_3_NO_6_)(C_4_H_6_N_2_)_2_]_*n*_, the carboxyl­ate groups of the 3-nitro­phthalate dianion ligand coordinate the Zn^II^ ion in a bis-monodentate mode. The Zn^II^ ion shows distorted tetra­hedral coordination as it is bonded to two O atoms from the carboxyl­ate groups of symmetry-related 3-nitro­phthalate anions and two N atoms of two independent 1-methyl­imidazole mol­ecules. The bridging 3-nitro­phthalate ligand allows the formation of one-dimensional chains in the *c* direction. The crystal structure is further stabilized by weak inter­molecular C—H⋯O hydrogen bonds.

## Related literature

For related structures with methyl­imidazole, see: Baca *et al.* (2003 ▶, 2004 ▶); Zhao (2008 ▶). For related coordination modes of phthalate and substituted phthalate with metal, see: Biagini Cingi *et al.* (1978 ▶); Guo & Guo (2007 ▶); Ma *et al.* (2004 ▶); Wang *et al.* (2009 ▶); Yang *et al.* (2003 ▶).
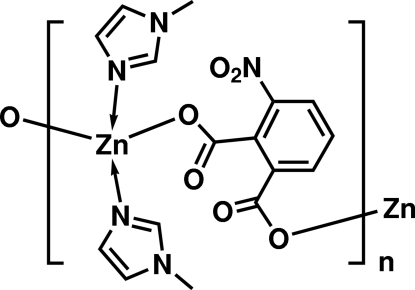


## Experimental

### 

#### Crystal data

[Zn(C_8_H_3_NO_6_)(C_4_H_6_N_2_)_2_]
*M* *_r_* = 438.70
Monoclinic, 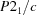

*a* = 8.375 (2) Å
*b* = 16.005 (4) Å
*c* = 14.057 (4) Å
β = 102.618 (4)°
*V* = 1838.7 (8) Å^3^
*Z* = 4
Mo *K*α radiation
μ = 1.38 mm^−1^
*T* = 294 K
0.18 × 0.06 × 0.06 mm

#### Data collection

Rigaku Saturn CCD area-detector diffractometer
Absorption correction: multi-scan (*CrystalClear*; Rigaku/MSC, 2005 ▶) *T*_min_ = 0.883, *T*_max_ = 0.921
13428 measured reflections
3240 independent reflections
2904 reflections with *I* > 2σ(*I*)
*R*_int_ = 0.027

#### Refinement

*R*[*F* ^2^ > 2σ(*F* ^2^)] = 0.028
*wR*(*F* ^2^) = 0.074
*S* = 1.06
3240 reflections
255 parameters
H-atom parameters constrained
Δρ_max_ = 0.43 e Å^−3^
Δρ_min_ = −0.37 e Å^−3^

### 

Data collection: *CrystalClear* (Rigaku/MSC, 2005 ▶); cell refinement: *CrystalClear*; data reduction: *CrystalClear*; program(s) used to solve structure: *SHELXS97* (Sheldrick, 2008 ▶); program(s) used to refine structure: *SHELXL97* (Sheldrick, 2008 ▶); molecular graphics: *SHELXTL* (Sheldrick, 2008 ▶); software used to prepare material for publication: *SHELXTL*.

## Supplementary Material

Crystal structure: contains datablock(s) I, global. DOI: 10.1107/S1600536812004576/bh2410sup1.cif

Structure factors: contains datablock(s) I. DOI: 10.1107/S1600536812004576/bh2410Isup2.hkl

Additional supplementary materials:  crystallographic information; 3D view; checkCIF report

## Figures and Tables

**Table 1:** Selected bond angles (°)

O2—Zn1—O3^i^	105.60 (6)
O2—Zn1—N3	123.39 (7)
O3^i^—Zn1—N3	110.17 (7)
O2—Zn1—N1	104.66 (6)
O3^i^—Zn1—N1	103.44 (7)
N3—Zn1—N1	107.78 (7)
O1—C1—O2	126.52 (18)
O4—C8—O3	125.73 (19)

Symmetry code: (i) 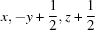.

**Table 2:** Hydrogen-bond geometry (Å, °)

*D*—H⋯*A*	*D*—H	H⋯*A*	*D*⋯*A*	*D*—H⋯*A*
C9—H9⋯O4	0.93	2.25	3.156 (3)	166
C11—H11⋯O1^ii^	0.93	2.53	3.297 (3)	140
C12—H12*A*⋯O5^iii^	0.96	2.44	3.078 (3)	124
C13—H13⋯O1^i^	0.93	2.46	3.373 (3)	169
C16—H16*B*⋯O2^iv^	0.96	2.44	3.345 (3)	158

Symmetry codes: (i) 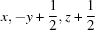; (ii) 
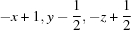
; (iii) 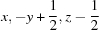; (iv) 
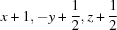
.

**Fig. 1.**
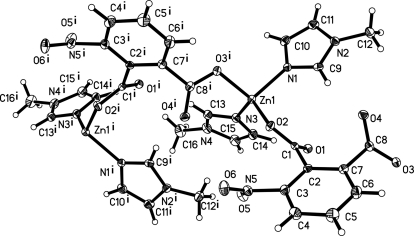
A view of the structure of the title complex, showing the coordination environment for Zn atom; displacement ellipsoids are drawn at the 30% probability level [Symmetry code: (i) x, -y+1/2, z+1/2].

**Fig. 2.**
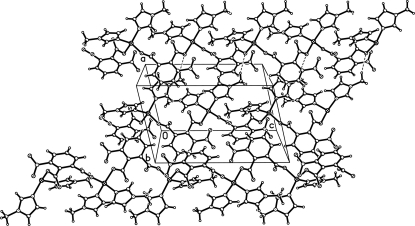
The packing diagram of the complex, viewed down the b axis, showing its one dimensional chain structure along the c direction.

## supplementary crystallographic information

### Comment

Aromatic dicarboxylate ligands such as phthalate (phth) and substituted phthalate have been used in architecture of polymeric metal complexes because they can act as a bis-monodentate, bis-bidentate and combined modes of coordination to form short bridges *via* one carboxylato end, or long bridges *via* the benzene ring, leading to a great variety of structures (Zhao, 2008; Biagini Cingi *et al.*, 1978; Guo & Guo, 2007; Wang *et al.*, 2009; Ma *et al.*, 2004; Baca *et al.*, 2003, 2004; Yang *et al.*, 2003). We have used the 3-nitrophthalate dianion as a ligand, and have obtained the title novel four-coordinate 3-nitrophthalate-zinc complex. We describe here the structure of this one-dimensional metal-nitrophthalate coordination polymer with bis-monodentate coordination mode.

The asymmetric unit in the structure of the title compound comprises one Zn atom, one complete 3-nitrophthalate dianion and two non-equivalent 1-methylimidazole molecules, and is shown in Fig. 1 in a symmetry-expanded view, which displays the full coordination sphere of the Zn atom. Selected geometric parameters are given in Table 1.

The Zn atom exhibits a distorted tetrahedral environment with atoms O2, O3^i^ (see Fig. 1 for symmetry codes) of two non-equivalent 3-nitrophthalate dianions and N1 and N3 atoms of coordinated 1-methylimidazole molecules (see Table 1 for bond lengths and angles), and this results in forming one-dimensional chains along the *c* direction. These are further aggregated into a three-dimensional framework *via* weak C—H···O interactions (see Table 2). A packing diagram is shown in Fig. 2.

### Experimental

Zinc oxide (0.21 g, 2.5 mmol) was added to a stirred solution of 3-nitrophthalic acid (0.53 g, 2.5 mmol) in boiling water (20.0 ml) over a period of 40 min, then drip 1-methylimidazole (0.33 g, 4 mmol) in the solution. After filtration, slow evaporation over a period of one week at room temperature provided colorless needle of the title complex.

### Refinement

The H atoms were treated as riding, with C—H = 0.93 Å and *U*_iso_ (H) = 1.2 *U*_eq_(C) for aromatic CH groups, and C—H = 0.96 Å and *U*_iso_ (H) = 1.5 *U*_eq_(C) for methyl CH_3_ groups.

### Crystal data

**Table:**

[Zn(C_8_H_3_NO_6_)(C_4_H_6_N_2_)_2_]	*F*(000) = 896
*M**_r_* = 438.70	*D*_x_ = 1.585 Mg m^−^^3^
Monoclinic, *P*2_1_/*c*	Mo *K*α radiation, λ = 0.71073 Å
Hall symbol: -P 2ybc	Cell parameters from 6551 reflections
*a* = 8.375 (2) Å	θ = 1.5–27.9°
*b* = 16.005 (4) Å	µ = 1.38 mm^−^^1^
*c* = 14.057 (4) Å	*T* = 294 K
β = 102.618 (4)°	Needle, colorless
*V* = 1838.7 (8) Å^3^	0.18 × 0.06 × 0.06 mm
*Z* = 4	

### Data collection

**Table:**

Rigaku Saturn CCD area-detector diffractometer	3240 independent reflections
Radiation source: rotating anode	2904 reflections with *I* > 2σ(*I*)
Confocal monochromator	*R*_int_ = 0.027
Detector resolution: 28.57 pixels mm^-1^	θ_max_ = 25.0°, θ_min_ = 2.0°
ω scans	*h* = −9→9
Absorption correction: multi-scan (*CrystalClear*; Rigaku/MSC, 2005)	*k* = −19→18
*T*_min_ = 0.883, *T*_max_ = 0.921	*l* = −16→16
13428 measured reflections	

### Refinement

**Table:**

Refinement on *F*^2^	Primary atom site location: structure-invariant direct methods
Least-squares matrix: full	Secondary atom site location: difference Fourier map
*R*[*F*^2^ > 2σ(*F*^2^)] = 0.028	Hydrogen site location: inferred from neighbouring sites
*wR*(*F*^2^) = 0.074	H-atom parameters constrained
*S* = 1.06	*w* = 1/[σ^2^(*F*_o_^2^) + (0.0448*P*)^2^ + 0.3182*P*] where *P* = (*F*_o_^2^ + 2*F*_c_^2^)/3
3240 reflections	(Δ/σ)_max_ = 0.009
255 parameters	Δρ_max_ = 0.43 e Å^−^^3^
0 restraints	Δρ_min_ = −0.37 e Å^−^^3^
0 constraints	

### Fractional atomic coordinates and isotropic or equivalent isotropic displacement parameters (Å^2^)

**Table:**

	*x*	*y*	*z*	*U*_iso_*/*U*_eq_	
Zn1	0.37841 (3)	0.207619 (14)	0.455381 (16)	0.01901 (10)	
O1	0.40470 (16)	0.33302 (8)	0.29849 (10)	0.0208 (3)	
O2	0.21557 (17)	0.27506 (9)	0.36867 (10)	0.0232 (3)	
O3	0.25521 (18)	0.36236 (9)	0.02871 (10)	0.0240 (3)	
O4	0.21554 (18)	0.24772 (9)	0.11179 (10)	0.0247 (3)	
O5	0.1941 (2)	0.44350 (11)	0.44209 (11)	0.0380 (4)	
O6	−0.0626 (2)	0.41913 (11)	0.43565 (12)	0.0429 (4)	
N1	0.4567 (2)	0.12574 (10)	0.36701 (12)	0.0205 (4)	
N2	0.4793 (2)	0.05951 (11)	0.23352 (12)	0.0242 (4)	
N3	0.5739 (2)	0.25540 (11)	0.54444 (12)	0.0230 (4)	
N4	0.7568 (2)	0.28401 (11)	0.67687 (13)	0.0282 (4)	
N5	0.0545 (2)	0.42875 (11)	0.39749 (13)	0.0289 (4)	
C1	0.2635 (2)	0.32439 (12)	0.30897 (13)	0.0178 (4)	
C2	0.1264 (2)	0.37573 (12)	0.24794 (14)	0.0180 (4)	
C3	0.0247 (3)	0.42461 (13)	0.29116 (15)	0.0246 (5)	
C4	−0.0997 (3)	0.47334 (15)	0.23928 (17)	0.0353 (6)	
H4	−0.1657	0.5046	0.2712	0.042*	
C5	−0.1245 (3)	0.47481 (16)	0.13908 (17)	0.0392 (6)	
H5	−0.2058	0.5085	0.1026	0.047*	
C6	−0.0277 (3)	0.42597 (14)	0.09312 (16)	0.0320 (5)	
H6	−0.0457	0.4265	0.0255	0.038*	
C7	0.0960 (2)	0.37607 (12)	0.14624 (14)	0.0205 (4)	
C8	0.1969 (2)	0.32282 (13)	0.09277 (14)	0.0208 (4)	
C9	0.4173 (2)	0.12626 (12)	0.27040 (15)	0.0217 (5)	
H9	0.3547	0.1675	0.2331	0.026*	
C10	0.5501 (3)	0.05407 (14)	0.39165 (16)	0.0283 (5)	
H10	0.5967	0.0371	0.4549	0.034*	
C11	0.5631 (3)	0.01286 (14)	0.31019 (16)	0.0302 (5)	
H11	0.6178	−0.0372	0.3066	0.036*	
C12	0.4611 (3)	0.03974 (15)	0.13012 (16)	0.0350 (6)	
H12A	0.3992	0.0830	0.0914	0.053*	
H12B	0.4050	−0.0126	0.1161	0.053*	
H12C	0.5673	0.0358	0.1150	0.053*	
C13	0.6139 (3)	0.24697 (13)	0.64004 (15)	0.0244 (5)	
H13	0.5510	0.2190	0.6768	0.029*	
C14	0.6993 (3)	0.30033 (14)	0.51958 (17)	0.0305 (5)	
H14	0.7046	0.3160	0.4566	0.037*	
C15	0.8124 (3)	0.31792 (15)	0.60038 (17)	0.0344 (6)	
H15	0.9094	0.3473	0.6039	0.041*	
C16	0.8332 (3)	0.29180 (16)	0.78106 (17)	0.0407 (6)	
H16A	0.8170	0.3474	0.8027	0.061*	
H16B	0.9483	0.2807	0.7910	0.061*	
H16C	0.7842	0.2524	0.8176	0.061*	

### Atomic displacement parameters (Å^2^)

**Table:**

	*U*^11^	*U*^22^	*U*^33^	*U*^12^	*U*^13^	*U*^23^
Zn1	0.02000 (15)	0.01878 (15)	0.01866 (14)	−0.00048 (9)	0.00515 (10)	0.00074 (9)
O1	0.0161 (8)	0.0221 (8)	0.0249 (7)	0.0007 (6)	0.0061 (6)	0.0011 (6)
O2	0.0216 (8)	0.0265 (8)	0.0231 (7)	0.0019 (6)	0.0087 (6)	0.0072 (6)
O3	0.0287 (8)	0.0241 (8)	0.0211 (7)	0.0016 (6)	0.0099 (6)	−0.0014 (6)
O4	0.0267 (9)	0.0216 (8)	0.0249 (8)	0.0037 (6)	0.0036 (6)	−0.0006 (6)
O5	0.0327 (10)	0.0483 (11)	0.0323 (9)	0.0025 (8)	0.0058 (7)	−0.0094 (8)
O6	0.0411 (11)	0.0552 (11)	0.0412 (10)	0.0013 (9)	0.0285 (8)	−0.0008 (9)
N1	0.0228 (10)	0.0190 (9)	0.0207 (9)	0.0013 (7)	0.0068 (7)	0.0010 (7)
N2	0.0248 (10)	0.0224 (9)	0.0269 (9)	−0.0005 (8)	0.0086 (8)	−0.0023 (8)
N3	0.0197 (9)	0.0255 (10)	0.0245 (9)	−0.0002 (7)	0.0066 (7)	−0.0014 (8)
N4	0.0187 (10)	0.0357 (11)	0.0295 (10)	0.0015 (8)	0.0036 (8)	−0.0062 (8)
N5	0.0340 (12)	0.0273 (10)	0.0289 (10)	0.0058 (8)	0.0146 (9)	−0.0006 (8)
C1	0.0217 (11)	0.0157 (10)	0.0163 (10)	−0.0006 (8)	0.0047 (8)	−0.0030 (8)
C2	0.0163 (10)	0.0169 (10)	0.0220 (10)	−0.0001 (8)	0.0066 (8)	0.0023 (8)
C3	0.0248 (12)	0.0261 (12)	0.0251 (11)	0.0029 (9)	0.0106 (9)	0.0013 (9)
C4	0.0301 (14)	0.0393 (14)	0.0395 (14)	0.0162 (11)	0.0142 (11)	0.0017 (11)
C5	0.0300 (14)	0.0484 (16)	0.0380 (14)	0.0215 (11)	0.0047 (11)	0.0079 (12)
C6	0.0279 (13)	0.0407 (14)	0.0257 (12)	0.0086 (10)	0.0027 (9)	0.0041 (10)
C7	0.0166 (11)	0.0221 (11)	0.0232 (10)	0.0011 (8)	0.0052 (8)	0.0009 (9)
C8	0.0165 (11)	0.0261 (12)	0.0175 (10)	0.0002 (9)	−0.0013 (8)	−0.0027 (9)
C9	0.0187 (11)	0.0176 (11)	0.0292 (11)	−0.0002 (8)	0.0058 (8)	0.0011 (9)
C10	0.0273 (13)	0.0291 (12)	0.0285 (11)	0.0057 (10)	0.0059 (9)	0.0088 (10)
C11	0.0305 (13)	0.0240 (12)	0.0375 (13)	0.0091 (10)	0.0104 (10)	0.0043 (10)
C12	0.0433 (15)	0.0348 (13)	0.0287 (12)	−0.0035 (11)	0.0115 (11)	−0.0080 (11)
C13	0.0206 (11)	0.0263 (12)	0.0269 (11)	0.0016 (9)	0.0066 (9)	−0.0010 (9)
C14	0.0286 (13)	0.0348 (13)	0.0313 (13)	−0.0064 (10)	0.0134 (10)	−0.0026 (10)
C15	0.0237 (13)	0.0396 (14)	0.0425 (14)	−0.0095 (10)	0.0129 (10)	−0.0082 (12)
C16	0.0277 (14)	0.0570 (17)	0.0324 (14)	0.0029 (11)	−0.0047 (10)	−0.0035 (12)

### Geometric parameters (Å, º)

**Table:**

Zn1—O2	1.9454 (14)	C2—C7	1.396 (3)
Zn1—O3^i^	1.9612 (14)	C3—C4	1.376 (3)
Zn1—N3	1.9841 (17)	C4—C5	1.378 (3)
Zn1—N1	2.0116 (17)	C4—H4	0.9300
O1—C1	1.231 (2)	C5—C6	1.383 (3)
O2—C1	1.279 (2)	C5—H5	0.9300
O3—C8	1.281 (2)	C6—C7	1.389 (3)
O4—C8	1.234 (2)	C6—H6	0.9300
O5—N5	1.223 (2)	C7—C8	1.511 (3)
O6—N5	1.226 (2)	C9—H9	0.9300
N1—C9	1.326 (3)	C10—C11	1.346 (3)
N1—C10	1.389 (3)	C10—H10	0.9300
N2—C9	1.341 (3)	C11—H11	0.9300
N2—C11	1.371 (3)	C12—H12A	0.9600
N2—C12	1.463 (3)	C12—H12B	0.9600
N3—C13	1.319 (3)	C12—H12C	0.9600
N3—C14	1.380 (3)	C13—H13	0.9300
N4—C13	1.335 (3)	C14—C15	1.340 (3)
N4—C15	1.373 (3)	C14—H14	0.9300
N4—C16	1.470 (3)	C15—H15	0.9300
N5—C3	1.462 (3)	C16—H16A	0.9600
C1—C2	1.516 (3)	C16—H16B	0.9600
C2—C3	1.390 (3)	C16—H16C	0.9600
			
O2—Zn1—O3^i^	105.60 (6)	C5—C6—H6	119.4
O2—Zn1—N3	123.39 (7)	C7—C6—H6	119.4
O3^i^—Zn1—N3	110.17 (7)	C6—C7—C2	120.04 (19)
O2—Zn1—N1	104.66 (6)	C6—C7—C8	119.29 (18)
O3^i^—Zn1—N1	103.44 (7)	C2—C7—C8	120.67 (17)
N3—Zn1—N1	107.78 (7)	O4—C8—O3	125.73 (19)
C1—O2—Zn1	118.44 (13)	O4—C8—C7	119.97 (18)
C8—O3—Zn1^ii^	114.34 (13)	O3—C8—C7	114.30 (17)
C9—N1—C10	105.16 (17)	N1—C9—N2	111.11 (18)
C9—N1—Zn1	125.92 (14)	N1—C9—H9	124.4
C10—N1—Zn1	128.74 (14)	N2—C9—H9	124.4
C9—N2—C11	107.74 (17)	C11—C10—N1	109.76 (19)
C9—N2—C12	126.27 (18)	C11—C10—H10	125.1
C11—N2—C12	125.99 (19)	N1—C10—H10	125.1
C13—N3—C14	105.86 (18)	C10—C11—N2	106.23 (19)
C13—N3—Zn1	126.48 (15)	C10—C11—H11	126.9
C14—N3—Zn1	127.59 (15)	N2—C11—H11	126.9
C13—N4—C15	107.49 (19)	N2—C12—H12A	109.5
C13—N4—C16	125.6 (2)	N2—C12—H12B	109.5
C15—N4—C16	126.8 (2)	H12A—C12—H12B	109.5
O5—N5—O6	124.51 (19)	N2—C12—H12C	109.5
O5—N5—C3	117.59 (18)	H12A—C12—H12C	109.5
O6—N5—C3	117.88 (19)	H12B—C12—H12C	109.5
O1—C1—O2	126.52 (18)	N3—C13—N4	110.9 (2)
O1—C1—C2	119.99 (17)	N3—C13—H13	124.6
O2—C1—C2	113.49 (17)	N4—C13—H13	124.6
C3—C2—C7	116.93 (18)	C15—C14—N3	109.3 (2)
C3—C2—C1	121.21 (17)	C15—C14—H14	125.3
C7—C2—C1	121.86 (17)	N3—C14—H14	125.3
C4—C3—C2	123.5 (2)	C14—C15—N4	106.4 (2)
C4—C3—N5	117.16 (19)	C14—C15—H15	126.8
C2—C3—N5	119.23 (18)	N4—C15—H15	126.8
C3—C4—C5	118.6 (2)	N4—C16—H16A	109.5
C3—C4—H4	120.7	N4—C16—H16B	109.5
C5—C4—H4	120.7	H16A—C16—H16B	109.5
C4—C5—C6	119.7 (2)	N4—C16—H16C	109.5
C4—C5—H5	120.2	H16A—C16—H16C	109.5
C6—C5—H5	120.2	H16B—C16—H16C	109.5
C5—C6—C7	121.2 (2)		
			
O3^i^—Zn1—O2—C1	−177.09 (13)	C4—C5—C6—C7	−0.9 (4)
N3—Zn1—O2—C1	55.12 (16)	C5—C6—C7—C2	−1.2 (3)
N1—Zn1—O2—C1	−68.28 (15)	C5—C6—C7—C8	179.1 (2)
O2—Zn1—N1—C9	7.11 (18)	C3—C2—C7—C6	2.2 (3)
O3^i^—Zn1—N1—C9	117.49 (17)	C1—C2—C7—C6	−178.04 (19)
N3—Zn1—N1—C9	−125.83 (17)	C3—C2—C7—C8	−178.14 (18)
O2—Zn1—N1—C10	−167.15 (17)	C1—C2—C7—C8	1.7 (3)
O3^i^—Zn1—N1—C10	−56.76 (19)	Zn1^ii^—O3—C8—O4	1.6 (3)
N3—Zn1—N1—C10	59.91 (19)	Zn1^ii^—O3—C8—C7	−177.52 (12)
O2—Zn1—N3—C13	121.96 (17)	C6—C7—C8—O4	−129.1 (2)
O3^i^—Zn1—N3—C13	−3.9 (2)	C2—C7—C8—O4	51.2 (3)
N1—Zn1—N3—C13	−116.05 (18)	C6—C7—C8—O3	50.1 (3)
O2—Zn1—N3—C14	−61.5 (2)	C2—C7—C8—O3	−129.6 (2)
O3^i^—Zn1—N3—C14	172.64 (17)	C10—N1—C9—N2	0.3 (2)
N1—Zn1—N3—C14	60.4 (2)	Zn1—N1—C9—N2	−175.08 (13)
Zn1—O2—C1—O1	1.6 (3)	C11—N2—C9—N1	0.3 (2)
Zn1—O2—C1—C2	−177.94 (12)	C12—N2—C9—N1	−179.79 (19)
O1—C1—C2—C3	−125.2 (2)	C9—N1—C10—C11	−0.7 (2)
O2—C1—C2—C3	54.5 (2)	Zn1—N1—C10—C11	174.44 (15)
O1—C1—C2—C7	55.0 (3)	N1—C10—C11—N2	0.9 (3)
O2—C1—C2—C7	−125.3 (2)	C9—N2—C11—C10	−0.7 (2)
C7—C2—C3—C4	−1.3 (3)	C12—N2—C11—C10	179.3 (2)
C1—C2—C3—C4	178.9 (2)	C14—N3—C13—N4	0.1 (2)
C7—C2—C3—N5	−178.00 (18)	Zn1—N3—C13—N4	177.23 (14)
C1—C2—C3—N5	2.2 (3)	C15—N4—C13—N3	−0.3 (2)
O5—N5—C3—C4	−127.4 (2)	C16—N4—C13—N3	175.7 (2)
O6—N5—C3—C4	50.9 (3)	C13—N3—C14—C15	0.1 (3)
O5—N5—C3—C2	49.5 (3)	Zn1—N3—C14—C15	−176.93 (16)
O6—N5—C3—C2	−132.1 (2)	N3—C14—C15—N4	−0.3 (3)
C2—C3—C4—C5	−0.7 (4)	C13—N4—C15—C14	0.4 (3)
N5—C3—C4—C5	176.1 (2)	C16—N4—C15—C14	−175.5 (2)
C3—C4—C5—C6	1.8 (4)		

Symmetry codes: (i) *x*, −*y*+1/2, *z*+1/2; (ii) *x*, −*y*+1/2, *z*−1/2.

### Hydrogen-bond geometry (Å, º)

**Table:**

*D*—H···*A*	*D*—H	H···*A*	*D*···*A*	*D*—H···*A*
C9—H9···O4	0.93	2.25	3.156 (3)	166
C11—H11···O1^iii^	0.93	2.53	3.297 (3)	140
C12—H12*A*···O5^ii^	0.96	2.44	3.078 (3)	124
C13—H13···O1^i^	0.93	2.46	3.373 (3)	169
C16—H16*B*···O2^iv^	0.96	2.44	3.345 (3)	158

Symmetry codes: (i) *x*, −*y*+1/2, *z*+1/2; (ii) *x*, −*y*+1/2, *z*−1/2; (iii) −*x*+1, *y*−1/2, −*z*+1/2; (iv) *x*+1, −*y*+1/2, *z*+1/2.
